# Induction of Heat Shock Protein 70 in Mouse RPE as an In Vivo Model of Transpupillary Thermal Stimulation

**DOI:** 10.3390/ijms21062063

**Published:** 2020-03-17

**Authors:** Mooud Amirkavei, Marja Pitkänen, Ossi Kaikkonen, Kai Kaarniranta, Helder André, Ari Koskelainen

**Affiliations:** 1 Department of Neuroscience and Biomedical Engineering, School of Science, Aalto University, 00076 Espoo, Finland; mooud.amirkavei@aalto.fi (M.A.); marja.pitkanen@aalto.fi (M.P.); ossi.kaikkonen@aalto.fi (O.K.); 2 Department of Clinical Neuroscience, Division of Eye and Vision, St. Erik Eye Hospital, Karolinska Institutet, 17177 Stockholm, Sweden; 3 Department of Ophthalmology, Institute of Clinical Medicine, University of Eastern Finland and Kuopio University Hospital, 70210 Kuopio, Finland; kai.kaarniranta@uef.fi

**Keywords:** transpupillary laser-induced heating, heat shock protein 70 (HSP70), retinal pigment epithelium (RPE), age-related macular degeneration (AMD), immunohistology, mouse

## Abstract

The induction of heat shock response in the macula has been proposed as a useful therapeutic strategy for retinal neurodegenerative diseases by promoting proteostasis and enhancing protective chaperone mechanisms. We applied transpupillary 1064 nm long-duration laser heating to the mouse (C57Bl/6J) fundus to examine the heat shock response in vivo. The intensity and spatial distribution of heat shock protein (HSP) 70 expression along with the concomitant probability for damage were measured 24 h after laser irradiation in the mouse retinal pigment epithelium (RPE) as a function of laser power. Our results show that the range of heating powers for producing heat shock response while avoiding damage in the mouse RPE is narrow. At powers of 64 and 70 mW, HSP70 immunostaining indicates 90 and 100% probability for clearly elevated HSP expression while the corresponding probability for damage is 20 and 33%, respectively. Tunel staining identified the apoptotic regions, and the estimated 50% damaging threshold probability for the heating (ED50) was ~72 mW. The staining with Bestrophin1 (BEST1) demonstrated RPE cell atrophy with the most intense powers. Consequently, fundus heating with a long-duration laser provides an approachable method to develop heat shock-based therapies for the RPE of retinal disease model mice.

## 1. Introduction

Protein homeostasis, including the maintenance of correct folding and timely degradation of proteins, is essential to the function of cells. In order to survive under continuous exposure to environmental factors detrimental to protein function, cells employ stress response aiming at the restoration and preservation of proteostasis. The heat shock response is one of the prosurvival activities in cells, whose principal aim is to increase the number of molecular chaperones to cope with enhanced protein misfolding and aggregation. The most studied chaperones are the heat shock proteins (HSPs) that provide a unique system to regulate traffic of newly synthetized proteins between cellular compartments, promote the refolding of misfolded proteins, and inhibit the formation of toxic protein aggregates. Once capacity of HSPs is exceeded, proteins undergo degradation by ubiquitin-proteasome system or by autophagy [1,2,3,4,5].

The induction of heat shock response has been proposed as a useful therapeutic strategy for age-related neurodegenerative diseases by promoting proteostasis and enhancing protective chaperon mechanisms [5,6]. Dry or nonexudative age-related macular degeneration (AMD) is disease of the retina with a neurodegenerative component. It is strongly associated with the degeneration and death of retinal pigment epithelium (RPE) cells. One of the main functions of the RPE cells is to maintain a balanced cellular homeostasis of the photoreceptors [7,8,9]. In advanced AMD, as the RPE cells are damaged, it subsequently affects the function of the photoreceptors, and impairs vision. The pathogenesis of dry AMD is not yet fully understood, but during the progression of the disease, different multicomponent aggregates accumulate in the RPE environment [10]. High oxidative stress in the retina, RPE damage due to reactive oxygen species, disturbed autophagy, chronic inflammation, and malfunctioning of the complement system have been associated with dry AMD [11,12,13,14,15]. In addition, aged tissues generally have weakened ability to maintain proteostasis [5,16]. Currently, there are no effective treatments to dry AMD.

Different transpupillary fundus heating approaches have been developed with the intention to treat retinal diseases, including AMD. In recent years, the focus has been directed to non-damaging treatments that aim at mild temperature increases, below the threshold of cellular damage to avoid detrimental effects for vision [17,18,19]. Previous studies have demonstrated that by transpupillary laser irradiation of the fundus, HSPs expression level can be elevated in the RPE [18,19,20,21,22], and it has been proposed that the procedure could lead to therapeutical effects through the induced heat shock response [18,19,23,24,25,26].

The most useful method for performing non-damaging retinal heating has not been entirely resolved. Previously, it has been suggested that applying micropulsed laser for fundus heating helps avoiding cellular damage of the neural retina [27,28,29]. On the other hand, it has been estimated that micropulse and continuous wave lasers would be similar when comparing the clinical effect [19,30], and although micropulsing could reduce the heat conduction to the surrounding tissues within the RPE layer, at the same time it would narrow down the therapeutic window [31]. The previous preclinical fundus heating studies aimed to trigger heat shock response have usually applied rabbits as animal models [18,20,21,31] or focused on RPE cells in vitro [23,24,32] while very few studies have applied mice [19]. Mice have small eye size with challenging optics, and under anesthesia, their lenses are prone to form reversible opacity [33,34]. Additionally, the application of any animal model compared to in vitro approaches complicates the experimental setting and introduces variation in the results due to inter-individual and local differences in pigmentation [35], the lack of information about the actual temperature rise in the RPE, and possible eye movements under anesthesia [36]. Nevertheless, studying fundus heating treatments with mice is vastly important due to their significant role in ocular research and the possibility to assess treatment effectiveness with genetic mouse models of retinal diseases. 

In this study, we investigated the heat shock response by measuring the expression levels of HSP70 in the mouse RPE. To achieve the heat shock response, we applied continuous-wave laser to the mouse fundus under anesthesia and examined the effects produced in the RPE cells by immunohistological methods. Our aim was to address whether it is possible to heat the mouse fundus reliably and induce heat shock response without permanent tissue damage, by a preset laser power. In addition, we investigated the relationships between the heating power, protein hydrolysis, oxidative stress level, HSP70 protein levels, the size of affected area, and morphology of the RPE. The principal objectives were to demonstrate the induction of heat shock response in mouse RPE cells through moderate laser-induced heat, to advance the development of controlled RPE heating treatment methods, and to propose a novel RPE atrophy model by laser overexposure.

## 2. Results

### 2.1. Whole-Tissue Protein Analysis after Laser Inductions

**Figure 1:**
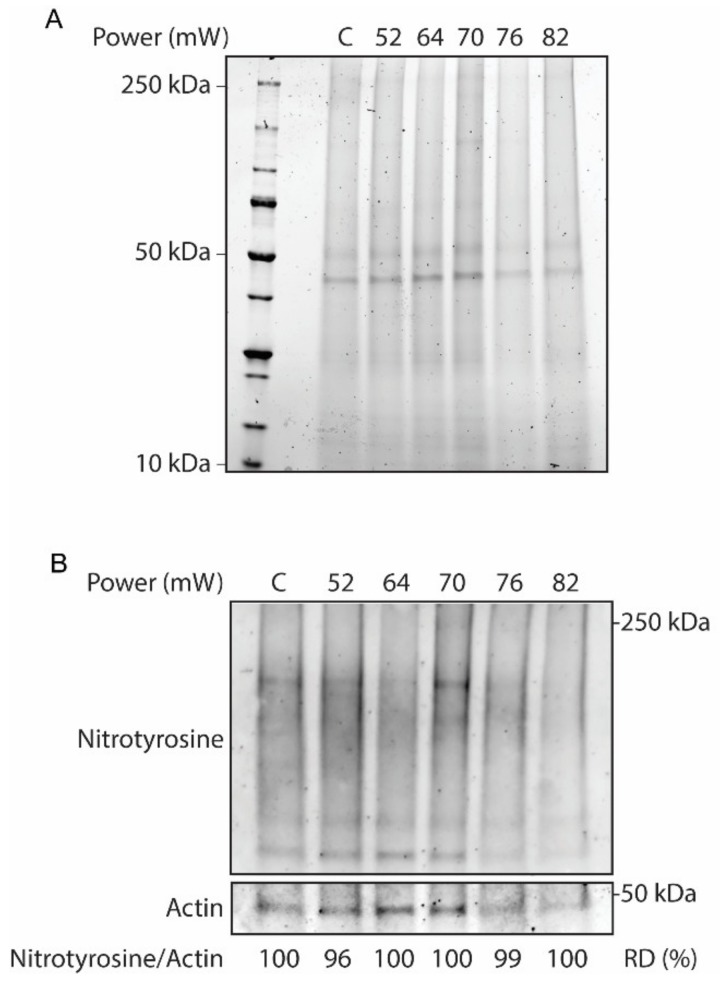
Whole-tissue protein extracts from laser-treated eyecups and non-lasered controls (C) across different laser powers. (**A**) SDS-PAGE stain-free gel to assess protein hydrolysis. (**B**) Western blot analysis for nitrotyrosine and actin as a loading control. The protein oxidative status was determined by relative densitometry (RD) in percentage of controls.

Validation of the laser on the overall tissue responses at different laser powers (52 mW, 64 mW, 70 mW, 76 mW, and 82 mW) at 24 h after laser irradiation was evaluated by Western blot. In this regard, protein analysis of RPE flatmount on stain-free SDS-PAGE was performed to visualize whole-protein content, as a hallmark of protein hydrolysis (Figure 1A). The SDS-PAGE results did not demonstrate discernable protein hydrolysis across the laser powers. Furthermore, the effect of the laser treatment on the activation of oxidative stress in the RPE flatmounts was investigated by Western blot for nitrotyrosine, a marker for pan-protein oxidation by nitric oxide mediated reactions by nitrosylation of tyrosine residues in proteins (Figure 1B). The immunoblot of nitrotyrosine antibody corrected against the loading control actin demonstrated no remarkable nitrosylation in response to the increasing powers of laser treatments, as confirmed by the relative densitometry of the immunoblots. 

### 2.2. Heat Shock Response

**Figure 2:**
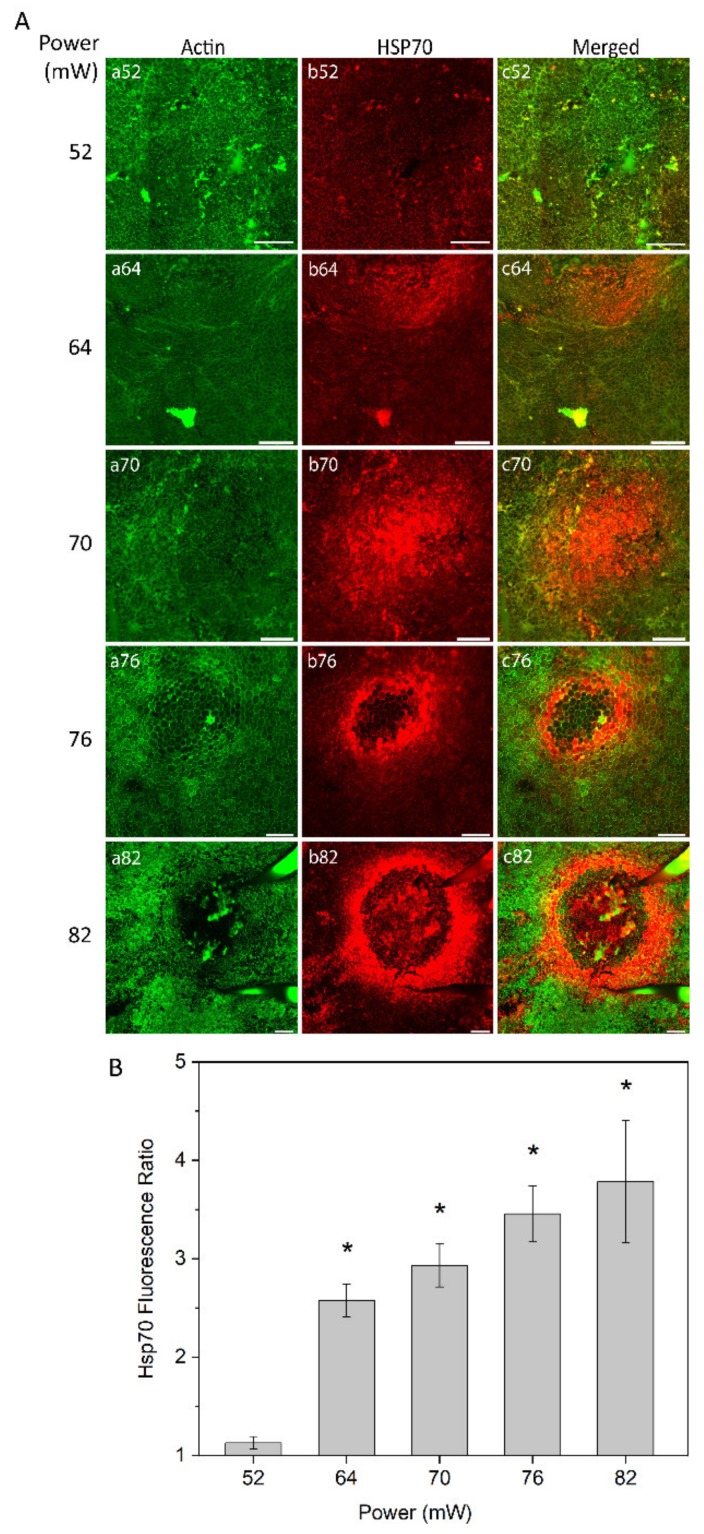
(**A**) heat shock protein (HSP) 70 expression in retinal pigment epithelium (RPE) flatmount across different laser powers. Panels (a) represent actin staining by phalloidin (green); panels (b) HSP70 staining (red). (**B**) Semi-quantitative analysis of HSP70 fluorescence ratio of laser irradiation for different powers. * *p* < 0.05, determined by one-way analysis of variance (ANOVA). Scale bar: 100 µm.

The heat shock response of RPE cells was monitored at different laser powers (52 mW, 64 mW, 70 mW, 76 mW, and 82 mW) as analyzed by immunostaining of RPE flatmount 24 h after laser irradiation (Figure 2A). Fluorescent-labelled phalloidin allowed to visualize the boundary of single RPE cells binding to polymerized actin filaments, which define a circumferential bundle encircling cells at the apical side of their lateral borders (Figure 2A, panel a). The pattern and intensity of HSP70 expression at the examined laser powers was determined by immunostaining (Figure 2A, panel b). For the laser power of 52 mW, no or at least a very weak increase in HSP70 expression was detected (Figure 2A, panel b52). With the laser powers of 64 mW and 70 mW, HSP70 expression was increased, showing the highest intensity in the center of the heated area (Figure 2A, panels b64 and b70). Heat shock response to laser powers at 76 mW and 82 mW showed a ring-shaped expression of HSP70 with very strong expression at the border of the heated area and a weak or negligible expression in the center of the laser spot (Figure 2A, panels b76 and b82).

**Figure 3:**
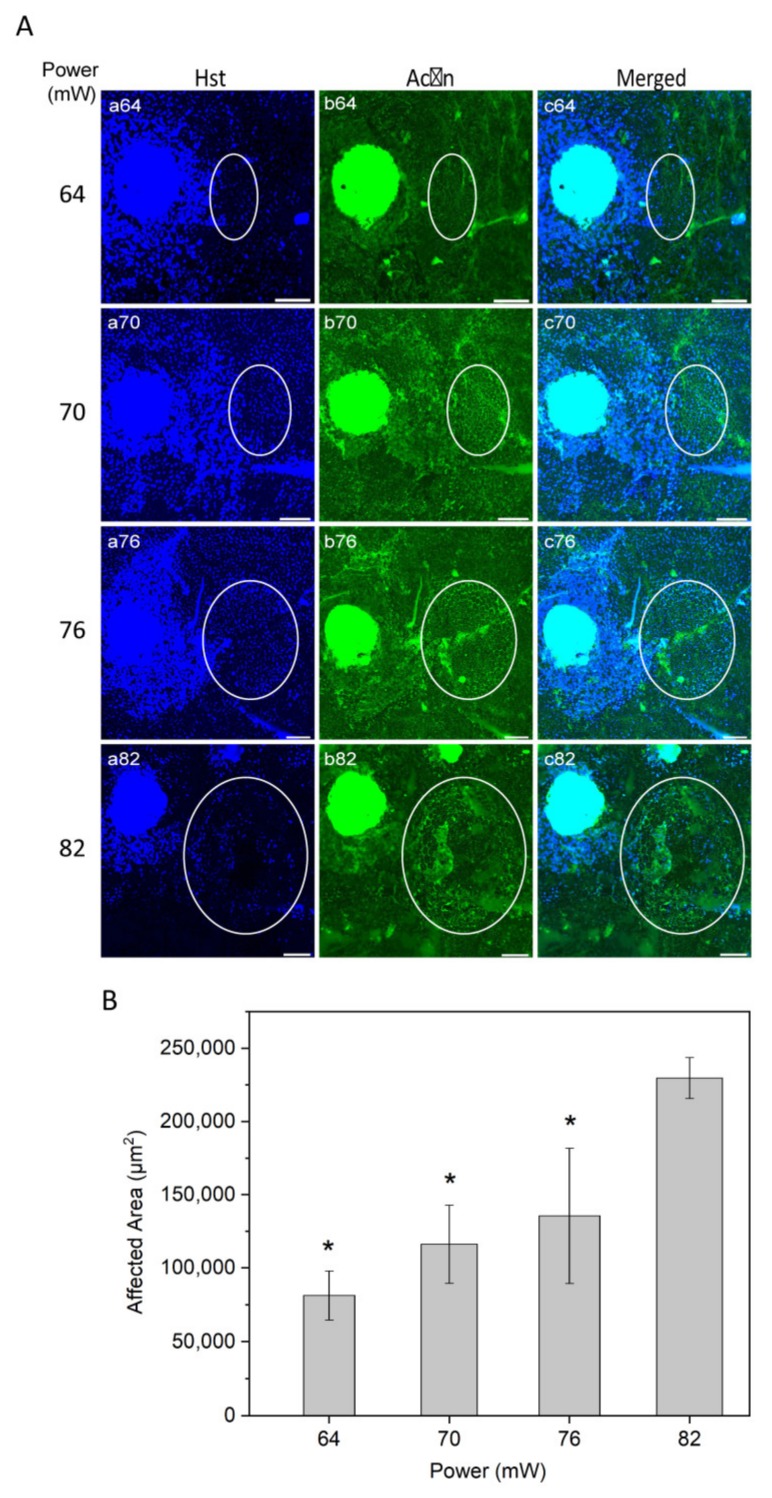
(**A**) Immunostaining of RPE flatmount for different laser powers. Panels (a) illustrate Hoechst (Hst) staining for nuclei (blue); panels (b) actin staining by phalloidin (green). White circles highlight the laser-induced area after irradiation. (**B**) Semi-quantitative analysis of laser-induced area after irradiation over different powers. * *p* < 0.05, determined by one-way ANOVA. Scale bar: 100 µm.

To obtain semi-quantitative analysis of the HSP70 expression at the area of induction, a local fluorescence intensity value of HSP70 for each treated eye was determined and normalized to the value of non-treated eye. The fluorescence intensity was defined for a constant area for each laser power, which increased proportionally to the affected area, as described below (see Figure 3). The relative value of HSP70 expression is shown in Figure 2B. The value of expression is very close to non-heated controls at 52 mW (approximate ratio of 1) and substantially increased at 64 mW. The HSP70 expression increased steadily with laser power elevation up to the highest power used (82 mW). The elevation of HSP70 expression was statistically significant (*p* < 0.05) at 64 mW, 70 mW, 76 mW, and 82 mW, as compared to 52 mW that were undistinguishable from controls.

### 2.3. The Area Affected by Laser Heating as a Function of Power

To evaluate the laser affected area as a function of power, nuclei staining with Hoechst 33258 and actin staining with phalloidin were examined (Figure 3A). Nuclei staining suggested that higher laser powers may cause damage to the RPE cells, resulting in a substantial nuclei loss at the highest power used (82 mW; Figure 3A, panel a82).

The laser impact on the structure of RPE cells, visually inspected from actin staining, increased gradually from non-discernible in non-heated controls at 52 mW, light at 64 mW, moderate at 70 mW, and intense at 76 and 82 mW. The representative images of actin stained RPE flatmount (Figure 3A, panel b) appeared without any morphologic alteration of RPE cells at powers of 64 and 70 mW.

The size of laser impact area was evaluated according to visual changes in the structure of the RPE cells and in the border of increased HSP70 expression (representative HSP70 data was presented in Figure 2). The affected area increased as a function of power (Figure 3B). The difference in the area was statistically significant at the powers 64, 70, and 76 mW compared to 82 (*p* < 0.05). A similar trend of affected area by laser power has been reported previously for histologically measured lesion widths, as well as Arrhenius and fixed-temperature models for retinal damage [19,37,38].

### 2.4. Apoptosis and RPE Viability Threshold

**Figure 4:**
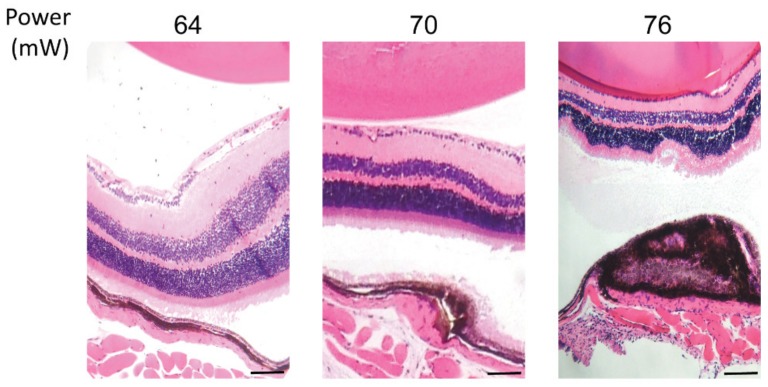
Hematoxylin and eosin staining of paraffin-embedded retinal cross sections of laser irradiations at different powers. Scale bar: 25 µm.

Considering the HSP70 expression threshold above 52 mW, hematoxylin and eosin (HE) staining of paraffin-embedded retinal cross sections was performed to visualize the effect of laser treatment on the retinal layers at powers of 64, 70, and 76 mW after 24 h post laser irradiation (Figure 4). The staining demonstrated no damage to retina cells at 64 mW, mild damage to RPE cells and choroid at 70 mW, and a strong damage to RPE cells and choroid, with observable damage to the neuroretina at 76 mW. In addition, a slight thermal fusion of the neuroretina to the RPE was observed at 76 mW during dissection of the tissue. Moreover, the electrophysiological function of neuroretina was examined by monitoring of the electroretinogram (ERG) from the eye receiving the treatment. The application of the laser at 82 mW did not induce considerable changes in the ERG flash response (see Figure S1 in Supplementary Material).

**Figure 5:**
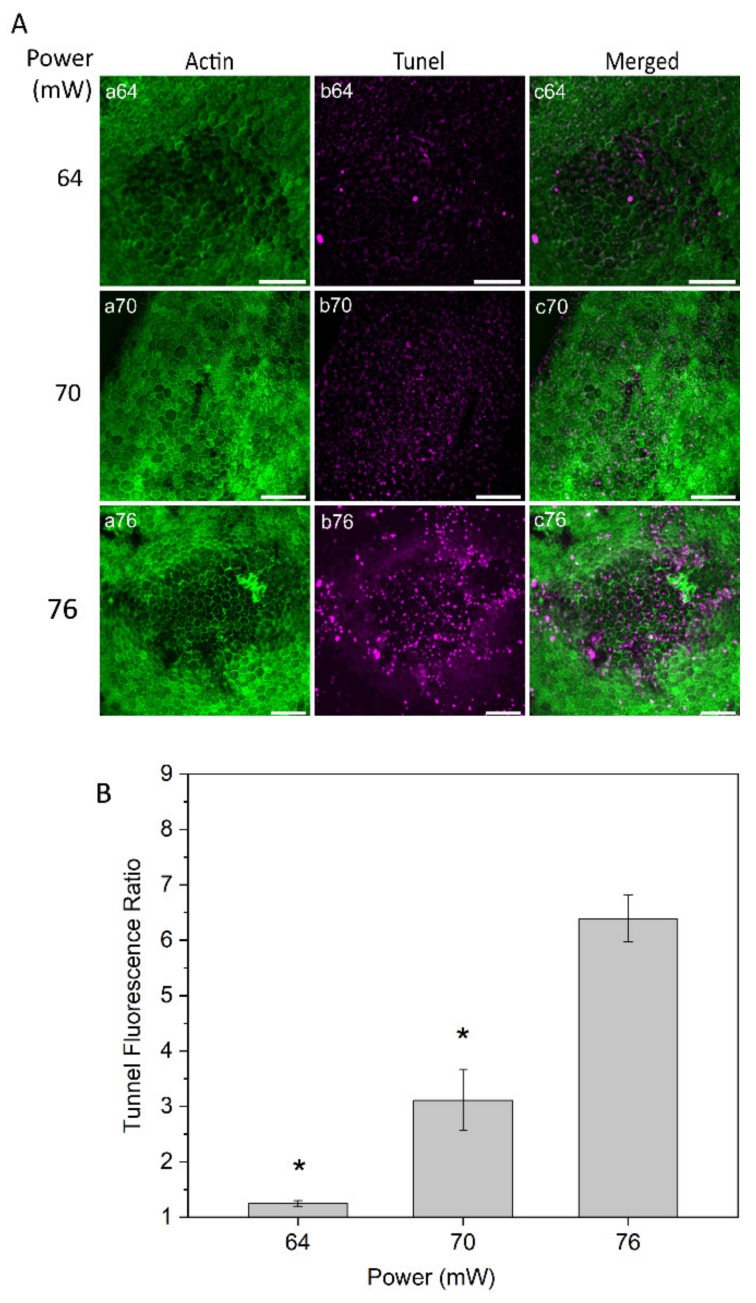
(**A**) Tunel staining of RPE flatmount for laser irradiations at different powers. Panels (a) displays actin staining by phalloidin (green), panels (b) represent Tunel staining (magenta). (**B**) Semi-quantitative analysis for Tunel fluorescence ratio of laser irradiation for different powers. * *p* < 0.05, determined by one-way ANOVA. Scale bar: 100 µm.

Tunel assay staining was conducted to evaluate cell damage at powers of 64, 70, and 76 mW 24 h after laser irradiation (Figure 5A). The RPE cell apoptosis results demonstrated no or very weak changes on the RPE cells with the power of 64 mW. With 70 mW, a weak apoptotic signal was observed in the center of the laser-treated area, suggesting mildly increased injury to RPE cells. More noticeable apoptotic staining was detected with the laser power of 76 mW (Figure 5A, panel b76). Subsequently, a semi-quantitative apoptosis analysis was performed, based on Tunel fluorescent intensity values and normalized to the value from the non-treated control eyes at a constant area for each power (Figure 5B). The analysis demonstrated a significant increase in RPE cell apoptosis at the power of 76 mW compared to the two other powers, 64 and 70 mW (*p* < 0.05).

**Figure 6:**
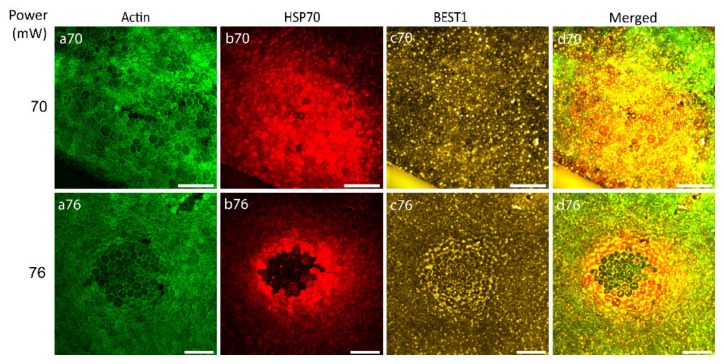
Immunostaining of RPE flatmount irradiated with different laser powers. Panels (a) represent actin staining by phalloidin (green), panels (b) HSP70 staining (red), panels (c) Bestrophin1 (BEST1) staining (yellow). Scale bar: 100 µm.

After the determination of the laser power threshold for RPE cell viability, the possibility of RPE atrophy generation caused by 70 and 76 mW laser irradiation was examined with Bestrophin1 (BEST1) staining (an integral membrane protein encoded by the Best1 gene localized predominantly to the basolateral membrane of RPE). Figure 6 illustrates that 24 h after laser irradiation with the laser power of 70 mW, no visible changes were observed indicating the non-atrophic naive RPE (Figure 6, panel c70), while the power of 76 mW generated an observable ring of BEST1 staining, suggestive of RPE atrophy (Figure 6, panel c76).

### 2.5. Estimation of Therapeutic Range based on Heat Shock Response and Cell Viability Results

**Figure 7:**
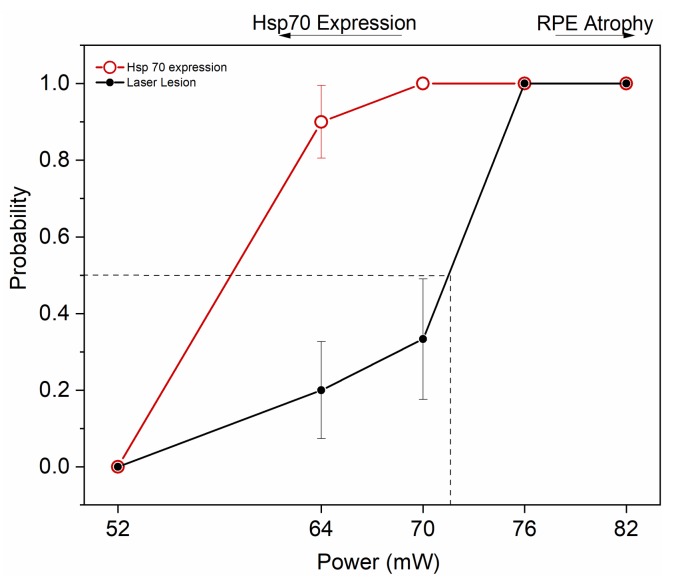
Probability analysis to estimate HSP70 expression threshold and ED50 damage threshold for 10 min duration of the 1064 nm laser to the mouse eye. Dashed line represents ED50. Zero represents no damage and no HSP70 expression, while 1 represents HSP70 expression and damage. 52 mW, *n* = 5; 64 mW, *n* = 10; 70 mW, *n* = 9; 76 mW, *n* = 7; and 82 mW, *n* = 5.

Figure 7 summarizes the results by illustrating the window of powers appropriate for HSP70 induction by transpupillary heating. By applying a heating power of 64 and 70 mW, 90 and 100% probability for clearly elevated HSP induction was determined, together with laser damage probability of 20 and 33%, respectively. The ED50 corresponding to 50% damage probability was at ~72 mW.

## 3. Discussion

The present study investigated the induction of heat shock response in wild-type C65Bl6 mouse RPE as an in vivo model for transpupillary laser-induced heating. The main goal was to estimate the laser power range that can be used to generate heat shock responses while avoiding damage to the RPE. In addition, the effects of the laser were assessed by further immunostaining examinations and by determining the size of the affected area. Thermal stimulation was produced by a considerably long duration and long-wavelength laser application to the mouse fundus.

Evaluation of protein hydrolysis and oxidative stress across the increasing laser powers demonstrated that there is no significant damage to the general protein content (Figure 1). Due to the focal nature of the laser irradiations, the assessment of HSP70 by immunostaining methods allows the correction by specific area, which is paramount in the methodology of focal heat shock response induction. The use of quantitative methods, such as Western blot or ELISA, would have been influenced by considerable tissue dilutions and could render the data biased. The expression of HSP70 was initially observed at 64 mW and increased at 70 mW. However, HSP70 expression at powers of 76 mW or more became ring-shaped, with a high expression ring around a dark center, likely due to cell damage (Figure 2). The ring-shaped area of HSP70 expression suggests that HSP70 around the damaged area is probably related to low RPE cell survival and apoptotic cell death. Thus, increasing the power above the damage threshold is assumed to increase the damaged area instead of enhancing the therapeutic heat shock responses by overexpression of HSP70. The trend of HSP70 expression at non-damaging to damaging irradiation is consistent with previous studies [18,19,23,24,31]. Semi-quantitative analysis of HSP70 levels as compared to control was analyzed to obtain insight into the local HSP70 expression.

RPE cell nuclei staining appeared normal for the powers of 64, 70, and 76 mW, while strong loss of RPE nuclei was observed at 82 mW. RPE actin staining displayed highly regular microfilaments in the form of hexagonal rings that define the apical boundaries of individual epithelia cells at 64 and 70 mW, while morphological changes by actin staining of RPE cells were apparent mildly and strongly at 76 and 82 mW, respectively. Consequently, the quantitative analysis of the size of the affected area versus applied laser power demonstrated an increasing correlation, as expected (Figure 3). In order to achieve further insight into the probability of damage by the used laser powers, HE and Tunel staining was examined. Irradiations with 1064 nm laser powers 64 to 70 mW (corresponding approximately to 230 to 250 mW/mm^2^, respectively) were considered non-damaging (Figure 4 and Figure 5) and the estimated damaging threshold to account for probability of 50% was at ~72 mW.

The assessment of BEST1 expression, as a marker for viable RPE cells, demonstrated differential expression in the RPE at the affected area of the laser irradiation and progressive RPE cell loss, a hallmark of geography atrophy leading to dry AMD (Figure 6). Albeit a careful analysis of RPE markers and RPE cells analysis is necessary, the powers above 76 mW with the laser presented here are expected to induce a novel RPE atrophy model [39]. Concomitantly, the results summarized in Figure 7 demonstrate that by applying a heating power of 64–70 mW, 90–100% probability for substantial HSP induction is obtained. However, with these laser powers, the damage probability is also elevated, amounting to about 20–33%. The probability for HSP induction and damage were 100% at 76–82 mW. Based on the present results, the useful range of laser heating powers to induce a heat shock response while avoiding damage in mouse RPE is narrow, and with clear HSP70 expression, non-zero probability for visible damage always remains. The possible factors affecting the repeatability of the heating treatment between individuals in mice include: variations in the RPE pigmentation levels between each mouse, as well as between different RPE areas; varying degrees of opacity development to the lens under anesthesia; and possible eye movements under anesthesia.

With regards to the heating outcome in terms of HSP induction and damage to the RPE displaying high levels of variation, a retinal temperature monitoring method could be employed. One possible approach for temperature monitoring may be provided by the determination of temperature-related changes in ERG responses. This method has been introduced as a proof of concept [40,41], and could be further developed for fundus heating protocols in mice by applying photopic focal ERG. Yet, the present laser heating setup did not enable the application of this approach. According to our results, by selecting laser powers close to 64 mW, the probability for damage would remain relatively low (~20%) and may be considered acceptable for single laser irradiations, particularly when taking into consideration the simplicity of the experimental protocol by applying a preset heating power, compared to the implementation of retinal temperature monitoring. However, with experimental protocols including repeated laser irradiations, temperature control of the heated area should be considered to reduce the risk of damage.

Analyses of the cellular response to the heat stimulation by assessing HSP70 induction serves two purposes. First, the HSP70 expression as a result of laser-induced heat provides feasibility to monitor and indicate thermal stimulation by laser irradiation. Secondly, the heat shock response, including increased HSP70 expression, is known as the only refolding system for misfolded protein, and it improves cellular survival under oxidative stress [1,42,43], a mechanism that has been directly associated with RPE cells.

Considering the role of HSP70 as the master regulator of the proteostasis network influencing the cytoprotection of cells, different studies have demonstrated its several functions, from repairing misfolded proteins to the regulation of lysosomal process, such as autophagy [5,44]. HSP70 has been shown to have a pivotal role in protein turnover in the pathogenesis of degenerative processes [44]. Importantly, a previous study shows that inducible HSP70 is a necessary component in degradation of protein aggregates by ubiquitin-proteasome system (UPS) through ubiquilin 2 (UBQLN-2) binding to aggregates, which is negligible under resting conditions but dramatically increased by heat shock [45]. HSP70 also affects the cellular redox status by modulating gluthatione-related enzyme antioxidant [46]. Furthermore, the pathway related to therapeutic effect of HSPs in treatments of chronic serous retinopathy and diabetic macular edema has been demonstrated [47,48,49]. The cell stress response has been found to stabilize adenylate-uridylate-rich elements of mRNA related to different proteins, such as vascular endothelial growth factor and Cox-2 [50,51], both associated with AMD. Non-damaging RPE thermal laser irradiation has been reported to promote the antioxidative potential by affecting the gluthatione balance in RPE cells [52].

In this regard, elucidation of the mechanistic basis of degenerative retinal disease, such as AMD, indicates a deficiency in cellular proteostasis [53,54]. The degenerative process is initiated by oxidative damage of proteins and phospholipids. It leads to the formation of cytotoxic protein aggregates in RPE cells, lipofuscin formation, accumulation of drusen deposits in the subretinal space, and inflammation [12,55], thus culminating in cell death [54]. However, HSP expression in general is decreased in the RPEs of human donor AMD eyes compared to healthy control eyes and the senescent RPE fails to induce HSP expression in response to oxidative damage [56,57,58]. HSP70 is expected to prevent the formation of cytotoxic protein aggregates, a hallmark of AMD, generated by the continuous oxidative stress in the RPE. Therefore, the present study deepens the knowledge that mild increase of HSP70 expression by non-damaging thermal stimulation in RPE cells may improve the cytoprotective capacity of RPE cells, and contribute to the prevention of retinal degenerative disorders.

## 4. Materials and Methods

### 4.1. Animals and Preparation

Animal procedures were approved by the Animal experiment Board of Finland and carried out in accordance with the ARVO statement for the use of Animal in Ophthalmic and Vision Research (Project code; ESAVI/6345/04.10.07/2015, approval date; 15th of September 2015). Three to five months old female mice of strain C57BL/6J (Envigo, Venray, Netherlands) were anesthetized with isoflurane (Animalcare, York, UK) in oxygen, and a subcutaneous injection of medetomidine (0.5 mg/kg, Orion Corporation, Espoo, Finland) was administered in order to prevent eye movements. The respiration was monitored with a piezoelectric sensor (MFi BV, Heerlen, Netherlands) and the isoflurane concentration was adjusted in the range of 1–2% to maintain the respiratory frequency between 2.0 and 2.5 Hz. The mouse was placed on a temperature-controlled polycarbonate platform equipped with anesthetic supply. The mouse’s head was stabilized with ear and forehead rubber grip bars. Pupillary dilation was achieved with topical administration of atropine sulfate (10 mg/mL, Chauvin, London, UK) and phenylephrine hydrochloride (100 mg/mL, Chauvin, London, UK) to the right eye of each mouse. Topical oxybuprocaine hydrochloride (4 mg/mL, Santen, Tampere, Finland) was used for local anesthesia of the cornea. During the experiment, methylcellulose solution (150 mg dissolved in 10 mL of 9 mg/mL NaCl) was applied to both eyes to prevent dehydration. The body temperature of the mouse was measured with a rectal thermistor (Betatherm 30K6A309I, Oy Farnell Finland Ab, Helsinki, Finland), and adjusted to 37.2 °C by controlling the temperatures of the air surrounding the animal and the water circulation inside the platform. After laser heating induction, isoflurane delivery was discontinued and a subcutaneous injection of atipamezole (0.25 mg/kg, Orion Corporation, Espoo, Finland) was administered to reverse sedative and analgesic effects of medetomidine.

### 4.2. Fundus Heating Laser Device and Heating Protocol

**Figure 8:**
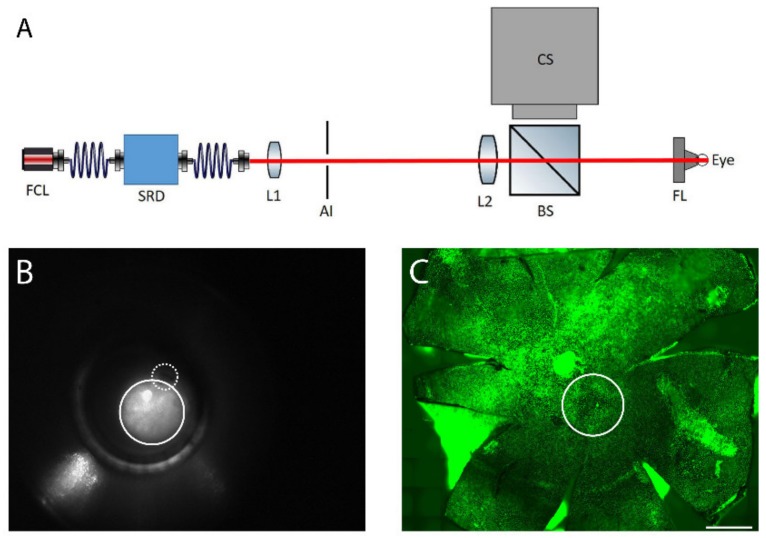
(**A**) The retinal laser treatment system: the output of the fiber-coupled laser system (FCL, λ = 1064 nm) is delivered through a speckle reduction device (SRD). The beam emitted from the speckle reduction device fiber is projected onto an adjustable iris (AI) by a lens (L1). The light passing through the iris is projected as a top hat spot onto the fundus by a second lens (L2) and a direct fundus lens (FL). Light scattered back from the eye is reflected towards a camera system (CS) by a beam splitter (BS). (**B**) Fundus image of mouse eye. The solid line circle represents the laser spot and the dashed-line shows the optic nerve. (**C**) Laser-induced spot in RPE flatmount is shown with solid circle, as identified by actin staining (green). Scale bar: 500 µm.

The fundus of the right eye was heated transpupillarily with a custom-made laser device (Figure 8A). The light from a fiber-coupled laser (FCL-1064 nm-4W, Changchun New Industries Optoelectronics Tech., Changchun, China) was directed through the optical system providing a top-hat profile beam of adjustable diameter and power. A direct fundus laser lens (Ocular 2mm Fundus Laser Lens, Ocular Instruments), attached to the laser device, was placed on the right cornea and the laser beam was focused through the lens onto the retina creating a spot size of approximately 600 µm, (Figure 8B,C). The device enabled imaging of the reflected laser light to assist in lens placement on the cornea and to indicate the location of the optic nerve in relation to the heating beam, if the optic nerve was inside the visible area (Figure 8B). An optical power meter (PM100D, Thorlabs Inc., New Jersey, USA) continuously monitored the heating power. A LabVIEW software controlled the laser power and treatment duration. For each animal, one of the laser powers of 52, 64, 70, 76, and 82 mW was applied. Exposure duration was standardized to 10 min as determined in preliminary experiments. Considering the approximate heating area (diameter of 600 µM), the calculated powers per area were 180, 230, 250, 270, and 290 mW/mm^2^. The left eye was used as a non-heated control.

### 4.3. Western Blot

For Western blotting, fixed-preserved eyecups of mice from 24 h post laser irradiation were used. Six eyecups for each laser power, including non-lasered controls, were suspended in 150 µL protein extraction buffer for fixed tissue (Qproteome FFPE Tissue Kit; Qiagen, Hilden, Germany). Tissues were homogenized by 3 × 10 s pulses at maximum speed with a VDI12 rotorstator (VWR, Dresden, Germany) and proceeded according to the manufacturer’s instructions. Whole-tissue protein extracts were quantified by a modified Bradford protein assay (Bio-Rad Laboratories, Hercules, CA, USA). Thirty micrograms of total protein extracts were separated by stain-free SDS-PAGE and transferred onto nitrocellulose membranes (Bio-Rad Laboratories). Blots were blocked using 1% casein in TBS (Bio-Rad Laboratories,). For primary antibodies: nitrotyrosine (sc-32757, 1:200; Santa Cruz Biotechnology, Paso Robles, CA, USA); and actin (1:2000, Sigma-Aldrich Corp. St. Louis, MO, USA) were incubated ON at 4 °C. Secondary antibodies anti-rabbit-Alexa Fluor 488 (1:1000, Invitrogen, Camarillo, MD, USA), and anti-rabbit-starbright Blue700 (1:1000, Invitrogen, Camarillo, MD, USA) were used for 1 h at RT. Both antibody steps were performed in 1% casein in TBS supplemented with 0.05% Tween-20 (Sigma-Aldrich Corp.), followed by extensive TBS washes. Visualization of bands was performed by ChemiDOC MP Imaging System (Bio-Rad Laboratories). Densitometry analysis were achieved using Image J software (National Institutes of Health, Bethesda, MD, USA).

### 4.4. Immunofluorescence

Immunofluorescence staining was performed to determine the HSP70 expression at powers of 52, 64, 70, 76, and 82 mW. Thereafter, in order to evaluate the capability of laser to induce a RPE atrophy model, BEST1 staining was examined at powers of 70 and 76 mW.

The mice were euthanized at 24 h after laser irradiation, corresponding to the time of maximum HSP70 expression [59], by CO_2_ inhalation and cervical dislocation. The eyes were enucleated and the posterior eyecups containing the RPE, choroid, and sclera were isolated as previously described [60]. Eyecups were fixed with 4% formaldehyde solution (FA; Solveco, Rosersberg, Sweden) in phosphate buffered saline (PBS) (Gibco, Paisley, UK) at room temperature (RT) for 20 min, permeabilized with 0,1% Triton X-100 (Sigma-Aldrich Corp.) in PBS, and antigen retrieval was achieved with Diva Decloaker (Biocare Medical, Concord, CA, USA) diluted to 1:10 and 3 min of microwave heating. Subsequently, samples proceeded to 1 h incubation at RT in 10% normal goat serum (Invitrogen, Camarillo, MD, USA) with 0.1% Triton X-100 (blocking solution). The primary antibodies, mouse monoclonal antibody to HSP70 (SPA-810, 1:50; Enzo Life Science, Santa Cruz Biotechnology, Paso Robles, CA, USA) or rabbit polyclonal antibody to BEST1 (1:200; Bioss, MA, USA), were diluted in blocking solution and incubated overnight at 4 °C. Incubation with secondary antibodies diluted in blocking solution was performed for 2 h at RT; Anti–rabbit-Alexa Fluor 594 (1:500, Invitrogen, Camarillo, MD, USA), Anti–donkey-Alexa Fluor 647 (1:500, Invitrogen, Camarillo, MD, USA), Anti-mouse-Alexa Fluor 350 (1:500, Invitrogen, Camarillo, MD, USA). Hoechst 33258 (5 g/L in PBS, Sigma-Aldrich Corp.) and Alexa Fluor 488-Phalloidin (Invitrogen, Camarillo, MD, USA) were added to secondary antibodies for counter-staining. Eyecups were extensively washed with PBS after all antibody steps, and post-fixed for 10 min in 4% FA-PBS at RT before flatmounting with fluorescent mounting medium (Dako, Carpinteria, CA, USA). Images were acquired with Axioimage fluorescence microscope with the Zen software (Zeiss, Gottingen, Germany).

### 4.5. RPE Cell Viability: Tunel Assay

A Terminal deoxynucleotidyl transferase-mediated dUTP nick-end labeling (Tunel) imaging assay kit (Clickit Plus Tunel 594, Thermo Fisher, USA) was used according to the manufacturer’s protocol to visualize apoptotic cells. Eyecups were prepared as for immunofluorescence. The RPE-side of the posterior eyecup flatmounts were imaged with an Axioimage fluorescence microscope with the Zen software (Zeiss, Gottingen, Germany).

### 4.6. Histology

Hematoxylin and eosin (HE) staining was applied to evaluate the effect of powers of 64, 70, and 76 mW on the retinal layers. After enucleation of the eye, the eyeballs were cleared from surrounding tissue and fixed in a formaldehyde-based buffer (FA; Solveco, Rosersberg, Sweden) for 24 h. Thereafter, the eyeballs were embedded in paraffin through the standard xylene method, and staining of sections of 4  µm with hematoxylin/eosin mix were performed using the Bond staining systems (Leica Biosystems, Newcastle, UK), according to the manufacturer’s instructions. Images were acquire on an Axioskop 40 microscope (Zeiss, Gottingen, Germany) coupled to a VisiCam TC10 (VWR, Lutterworth, UK).

### 4.7. Data and Statistical Analysis

For immunoblots in Figure 1, descriptive statistics were based on the ratio of the nitrotyrosine bands over the actin loading control, presented as percent of non-lasered controls. Semi-quantitative data are presented as mean ± standard error of the mean (SEM). For Figure 7, the samples were classified as positive or negative in terms of elevated HSP70 expression and observable damage. The proportion value was calculated by dividing the number of positive HSP70 expression or the number of positive visible damage with the total number of replicates for each specific power. Thereafter, results were presented as proportion (interpreted as probability) ± standard error of proportion (SEP). The number of biological repetitions were: 52 mW, *n* = 5; 64 mW, *n* = 10; 70 mW, *n* = 9; 76 mW, *n* = 7; and 82 mW, *n* = 5. Statistical analyses were calculated using IBM SPSS Statistics software version 21 (IBM, Armonk, NY, USA). All data were subjected to one-way analysis of variance (ANOVA), followed by Tukey’s test and Bonferroni corrected post-hoc tests for multiple comparison (*p*-values < 0.05 were considered significant).

## 5. Conclusions

Our transpupillary laser-mediated technology enables heating of the mouse fundus in vivo to induce heat shock response in the RPE. The setup enables the examination of heating treatment in one eye with the fellow-eye acting as a non-treated control. Fundus heating with a long-duration laser provides a potential method to develop heat shock-based therapies for the RPE of retinal disease model mice. The induction of HSP70 expression may lead to reduced levels of cytotoxic proteins and aggregates in the RPE cells by the activation of the proteostasis mechanisms in the region of interest. Further investigations to understand the cellular responses and therapeutic mechanisms of thermal stimulation in RPE are essential to elaborate the clinical translational or laser-mediated heat shock responses in ophthalmic diseases.

## Supplementary Materials

Supplementary materials can be found at https://www.mdpi.com/1422-0067/21/6/2063/s1. Figure S1: Electroretinogram recordings.

## Abbreviations

AMD	Age-related macular degeneration
ANOVA	Analysis of variance
BEST1	Bestrophin1
BS	Beam splitter
CS	Camera system
ED50	Estimated threshold corresponding to 50% probability of damage
FCL	Fiber-coupled laser
FA	Formaldehyde
Hst	Hoechst
HSPs	Heat shock proteins
HSP70	Heat shock protein 70
HE	Hematoxylin and eosin
PBS	Phosphate buffered saline
RPE	Retinal pigment epithelium
RT	Room temperature
SRD	Speckle reduction device
TUNEL	Terminal deoxynucleotidyl transferase-mediated dUTP nick-end labeling
UPS	Ubiquitin-proteasome system
UBQLN-2	Ubiquilin 2
