# Supplementary material for: Induction of Heat Shock Protein 70 in Mouse RPE as an In Vivo Model of Transpupillary Thermal Stimulation
Source: Int J Mol Sci. 2020 Mar 17;21(6):2063. doi: 10.3390/ijms21062063 (PMC7139698; doi:10.3390/ijms21062063)
Supplement: Supplementary file 1 [file ijms-21-02063-s001.pdf]

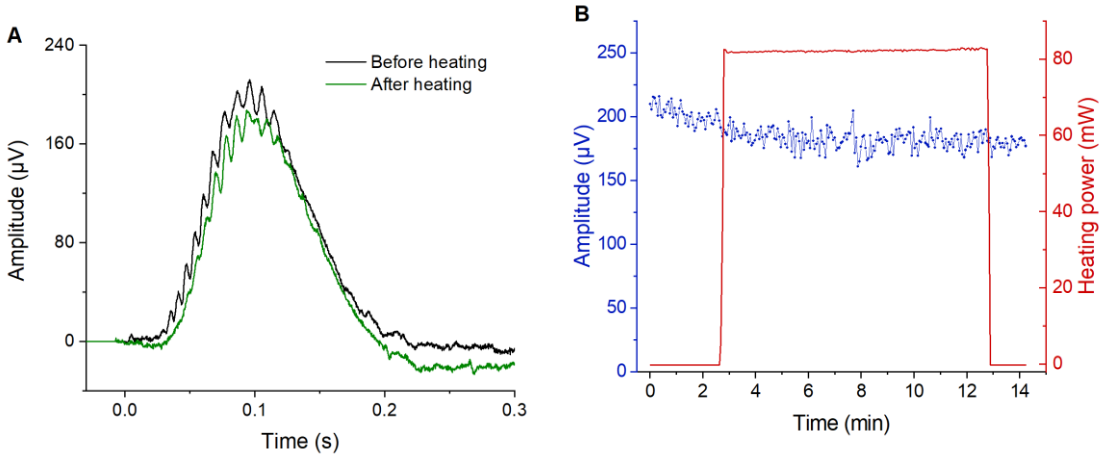

Figure 1S. **(A)** Presents the ERG responses to a brief dim flash stimulus recorded before and after 10 minutes of heating with 82 mW power. The ERG response exists and is clearly visible after the heating demonstrating the general function of the neural retina (green trace, averaged from 8 repetitions). The amplitude is only slightly reduced compared to the corresponding ERG response recorded before the heating (black trace, averaged from 8 repetitions). **(B)** Illustrates the behavior of the ERG amplitude as a function of time from the same experiment as panel A. The initiation and the termination of heating do not cause any sudden changes in the amplitude. Only a slight decrease in amplitude is visible, which is a common observation in ERG recordings and cannot be attributed to the heating.

The electroretinogram (ERG) was recorded with three Ag/AgCl pellet electrodes (EP1, World Precision Insruments Ltd., Hitchin, UK). The recording electrode was placed in a holder contacting the right cornea through a glass capillary (OD 1.5 mm ID 1.17 mm). The tip of the capillary was located next to the curved surface of the fundus laser lens. The reference electrode was located inside a holder constructed from a 1ml syringe. The tip of the syringe was placed around the left eye. Both electrode holders were filled with methylcellulose solution to provide electrical contact between the electrode and the cornea. The ground electrode was moisturized with the methylcellulose solution and placed in the rectum. To produce ERG responses, dim 60 µs flashes were delivered with a green LED (M530F2, Thorlabs Inc., New Jersey, USA). The LED was included in the heating device introduced in section 4.2. The device did not enable the application of full-field stimulus. Therefore, the exact size and intensity profile of the ERG stimulus light at the level of neural retina was not entirely defined, but it included both the heated area as well as some retinal area around it. The ERG signal was amplified 1000x, low-pass filtered (8-pole Bessel filter with fc = 1 kHz), and digitized at 5 kHz with 30 nV resolution.
